# Tracheal bifurcation repair for blunt thoracic trauma in a patient with COVID-19

**DOI:** 10.1186/s40792-023-01695-8

**Published:** 2023-06-15

**Authors:** Shoji Kuriyama, Kazuhiro Imai, Kasumi Tozawa, Shinogu Takashima, Ryo Demura, Haruka Suzuki, Yuzu Harata, Tatsuki Fujibayashi, Sumire Shibano, Yoshihiro Minamiya

**Affiliations:** Department of Thoracic Surgery, Akita University Graduate School of Medicine, 1-1-1 Hondo, Akita, 010-8543 Japan

**Keywords:** Tracheobronchial injury, Extracorporeal membrane oxygenation, COVID-19

## Abstract

**Background:**

Tracheobronchial injury (TBI) is a rare but potentially life-threatening trauma that requires prompt diagnosis and treatment. We present a case in which a patient with COVID-19 infection was successfully treated for a TBI through surgical repair and intensive care with extracorporeal membrane oxygenation (ECMO) support.

**Case presentation:**

This is the case of a 31-year-old man transported to a peripheral hospital following a car crash. Tracheal intubation was performed for severe hypoxia and subcutaneous emphysema. Chest computed tomography showed bilateral lung contusion, hemopneumothorax, and penetration of the endotracheal tube beyond the tracheal bifurcation. A TBI was suspected; moreover, his COVID-19 polymerase chain reaction screening test was positive. Requiring emergency surgery, the patient was transferred to a private negative pressure room in our intensive care unit. Due to persistent hypoxia and in preparation for repair, the patient was started on veno-venous ECMO. With ECMO support, tracheobronchial injury repair was performed without intraoperative ventilation. In accordance with the surgery manual for COVID-19 patients in our hospital, all medical staff who treated this patient used personal protective equipment. Partial transection of the tracheal bifurcation membranous wall was detected and repaired using 4-0 monofilament absorbable sutures. The patient was discharged on the 29th postoperative day without postoperative complications.

**Conclusions:**

ECMO support for traumatic TBI in this patient with COVID-19 reduced mortality risk while preventing aerosol exposure to the virus.

**Supplementary Information:**

The online version contains supplementary material available at 10.1186/s40792-023-01695-8.

## Background

Tracheobronchial injury (TBI) after blunt thoracic trauma is rare but often life-threatening, and appropriate treatment strategies are required. On the other hand, with emergency operations for patients with COVID-19, adequate preparation is mandatory to minimize infection spread. We present a case in which a patient with COVID-19 infection was successfully treated for a TBI through surgical repair and intensive care with extracorporeal membrane oxygenation (ECMO) support.

## Case presentation

**Fig. 1:**
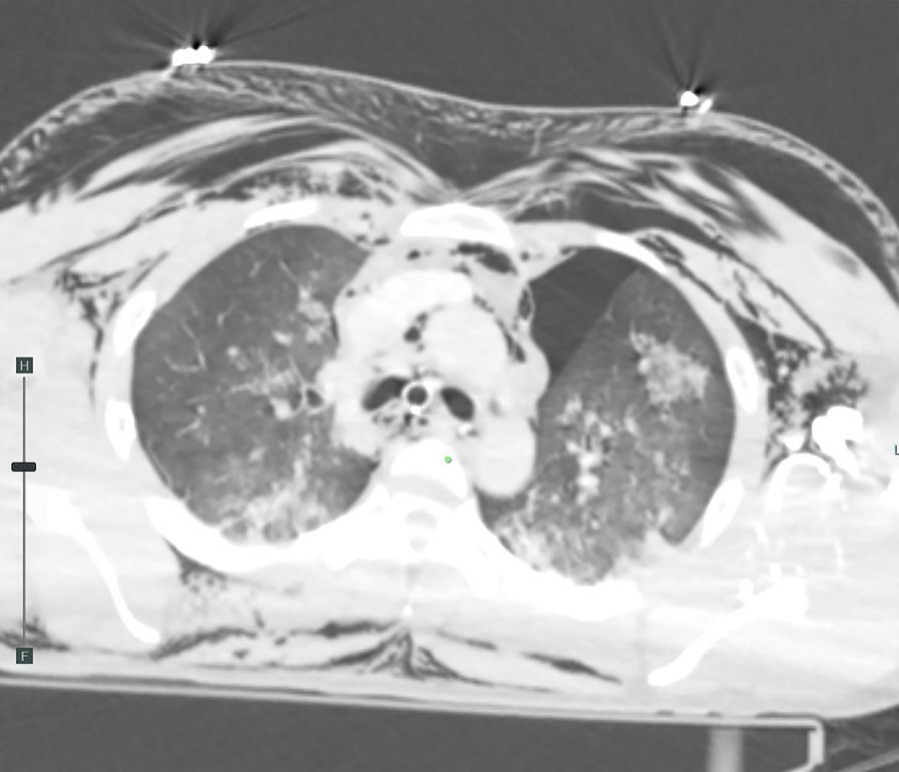
Computed tomography images showed the endotracheal tube inserted beyond the tracheal bifurcation

**Fig. 2:**
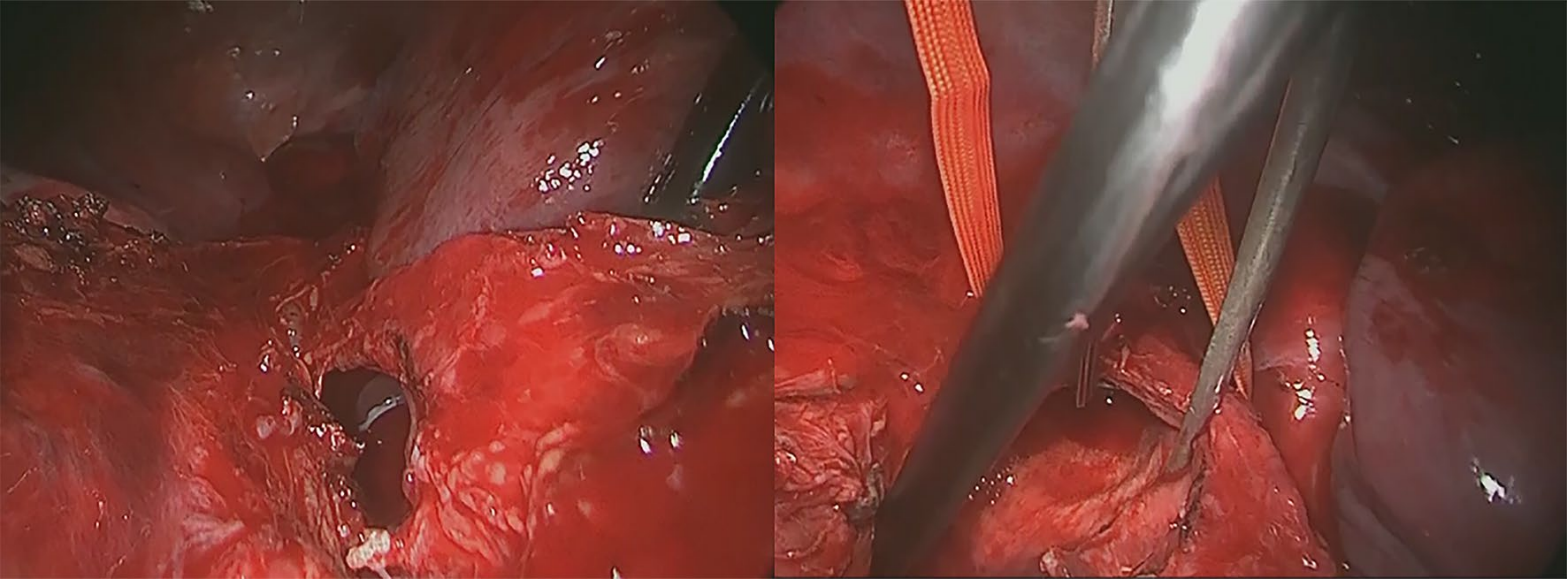
Intraoperative pictures showing partial transection of the membranous tracheal wall (**A**) and laceration of the right main bronchus wall (**B**)

**Fig. 3:**
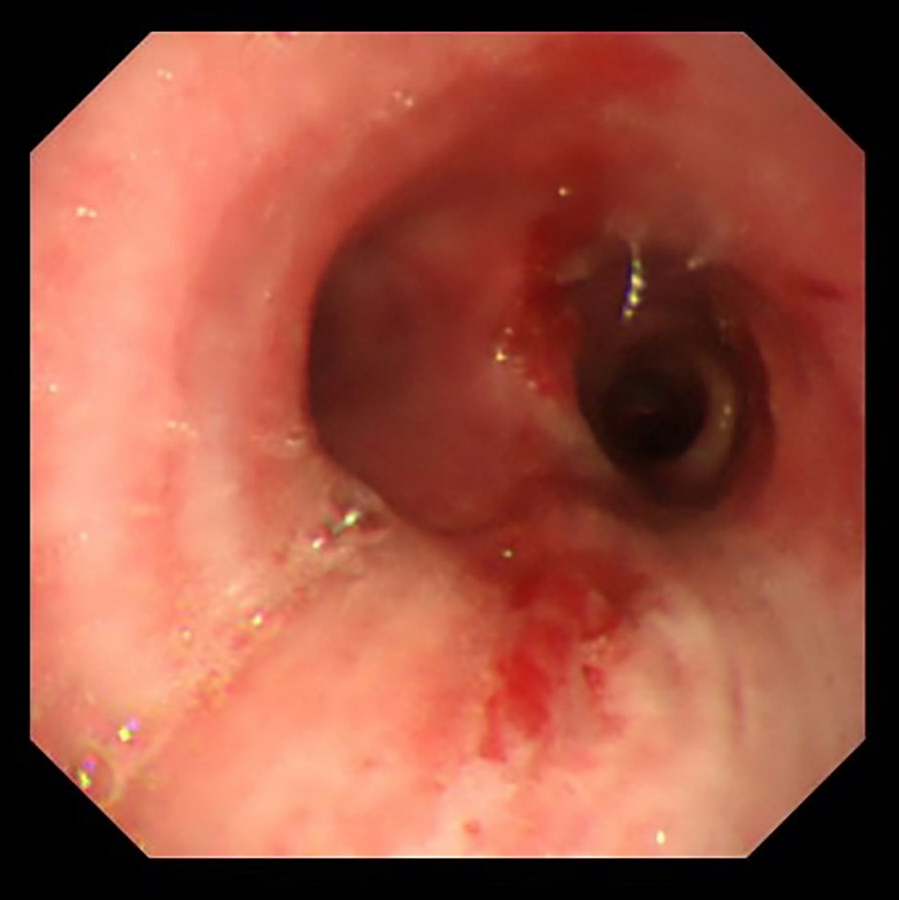
Image of bronchoscopy on the 24th postoperative day showing favorable healing process

A 31-year-old man was transported to a peripheral hospital with blunt thoracic trauma caused by a car crash. Tracheal intubation was performed without bronchoscopic guidance for severe hypoxia and subcutaneous emphysema. On whole-body computed tomography, bilateral pulmonary contusions and hemothorax were observed. In addition, the endotracheal tube was penetrated beyond the tracheal bifurcation and into the mediastinal space, thus CT raised suspicion of TBI (Fig. 1). There was no extravasation suggestive of active bleeding in other organs. Laboratory data showed a decrease in hemoglobin concentration (12.9 g/dL) and platelet count (10.6 × 10^4^). While the prothrombin time (PT) was within the normal range (13.6 s), the activated partial thromboplastin time (APTT) was prolonged (67.3 s), and there was an elevation in fibrin/fibrinogen degradation products (FDP) (35.00 μg/mL) and D-dimer (16.48 μg/mL). In addition, the patient’s rapid COVID-19 antigen test and COVID-19 polymerase chain reaction (PCR) screening test was positive. Having determined that he required emergency surgery, the patient was transferred to a private negative pressure room in our intensive care unit (ICU). To facilitate the tracheal suture and prevent aerosol exposure, veno-venous ECMO support was implemented. The right femoral vein and right internal jugular vein were each cannulated with a 21-Fr cannula. Once the veno-venous ECMO circuit was flowing at 3.4 L/min (FiO2, 80%; sweep, 4 L/min), the patient was weaned from the ventilator. Heparin was not administered as a bolus injection or continuous intravenous infusion because of prolonged APTT due to trauma-induced coagulopathy, and the patient received 2 units of fresh frozen plasma. Following ECMO was established, bronchoscopy (disposable scope) was performed, and the endotracheal tube was adjusted into the tracheal lumen. The persistent mild bleeding hindered a detailed evaluation, but only laceration in the cartilaginous wall was observed. Left-sided bronchial injury was not seen. With ECMO support, surgical repair was performed without intraoperative mechanical ventilation. In accordance with the surgery manual for COVID-19 patients in our hospital, all medical staff who treated this patient donned personal protective equipment (Additional file 1: Fig. S1). The perioperative management is summarized in Additional file 2: Table S1. After a standard posterolateral thoracotomy in the right fifth intercostal space, partial transection of the membranous wall at the tracheal bifurcation and two-thirds circumferential laceration of the right main bronchus were also noted (Fig. 2). Trimming of the injured tracheal wall was not necessary as it was fresh tissue, and the lacerations were repaired by continuous running sutures using 4-0 monofilament absorbable sutures (PDS-II, Ethicon, Tokyo, Japan). The cartilage wall was sutured intraluminally, while the membranous wall was done extraluminally. A sixth intercostal muscle flap was buttressed over the tracheobronchial repair site. On the second postoperative day (POD), APTT improved to 40.4, prompting the initiation of systemic heparin anticoagulation. On the 10th POD, the patient was decannulated from ECMO, and left femoral vein thrombosis was detected on follow-up enhanced CT. Systemic heparin anticoagulation was initiated. He came off the ventilator on the 15thPOD. On the 23rd POD, thrombosis regression was observed on the following CT. A Follow-up bronchoscopy performed on the 24th POD showed good healing at the bronchial repair site (Fig. 3). The patient was discharged on the 29th POD. All medical staff who treated this patient were not infected with COVID-19.

## Discussion

TBI is defined as an injury between the cricoid cartilage and the division of the lobar bronchi into their segmental branches [1]. The causes of TBI include blunt trauma, explosion injuries, gunshot or other penetrating wounds, and iatrogenic injuries. Most TBIs are caused by motor vehicle accidents and are located within 2 cm of the carina [2]. Three possible mechanisms for TBI caused by blunt trauma have been proposed: (1) chest compression along the anterior–posterior axis, (2) a rapid increase in airway pressure caused by reflex closure of the glottis, and (3) shear forces at the carina caused by rapid deceleration such as occurs in motor vehicle accidents [2]. In the present case, it is most likely that shear forces associated with the car crash caused partial transection of the carina. Tracheobronchial injury is difficult to diagnose due to atypical clinical symptoms. In general, chest X-ray often show pneumothorax, subcutaneous emphysema, and mediastinal emphysema, but these findings are not specific to its injury. CT scan detects airway lesions with a sensitivity of 85% in hemodynamically stable patients [3]. Bronchoscopy is the most valuable method for the evaluation of airway injuries including site, nature, and extent of injury, and it should be considered when TBI is suspected. Although TBI occurs in less than 1% of cases of blunt thoracic trauma [4], early recognition and appropriate surgical intervention are essential because of the high mortality rate.

The treatments for TBIs include both surgical repair and conservative measures. Only patients with stable hemodynamics and small lacerations (< 4 cm) not involving all layers, in whom an orally inserted cuff can be positioned below the level of injury, and who exhibit no other major associated injuries can improve with conservative treatment [5]. Even in those cases, however, within 2–6 weeks after injury, the bronchus can become obstructed or stenosed due the development of granulation tissue [2]. Consequently, most TBIs require early surgical repair. In the present case, surgery was indicated due to persistent hypoxia and CT images suggesting the presence of a TBI involving all layers of the bronchus. The surgical approach for TBI repair depends on the location of the injury. Right posterolateral thoracotomy is the best approach to access the carina, the lower portion of the intrathoracic trachea, the right main bronchus, and the proximal 2 cm of the left bronchus [5]. In our case, a right posterolateral thoracotomy through the fifth intercostal space was used.

ECMO plays a key role in the management of complicated tracheobronchial surgical procedures and thereby reduces mortality risk [6]. In addition, the use of ECMO during airway surgery can avoid aggressive ventilation with endotracheal cuffed tubes, improve visualization of the surgical field, and support complex operative procedures under stable conditions [7]. In the present case, ECMO was rapidly instituted to improve oxygenation and prepare for surgical repair, which led to a favorable outcome. When introducing ECMO, anticoagulation is required to prevent circuit occlusion. However, severe trauma patients may present with traumatic brain injury, active bleeding, and coagulation disorders, necessitating a cautious determination of its suitability. The anticoagulation strategy for ECMO in trauma patients is not standardized, but target ranges for activated clotting time (ACT) of 180–220 s and APTT of 40–80 s are commonly set [8]. In the present case, ECMO without anticoagulation was started due to prolonged APTT with traumatic tissue damage, but the patient received heparin systemic anticoagulation on the second POD as the APTT improved. Anticoagulation during ECMO in trauma patients should be tailored based on continuous evaluation of individual coagulation parameters.

COVID-19 is a highly infectious disease caused by the SARS-CoV-2 virus, which can spread through contact and respiratory and airborne transmission. Therefore, medical staff must protect themselves by donning the appropriate personal protective equipment. At our institution, all transported patients undergo routine COVID-19 PCR tests or rapid antigen tests upon arrival at the emergency department, regardless of symptoms. In addition, all medical staff take sufficient precautions in accordance with our hospital's COVID-19 infection prevention manual while providing treatment. In addition, special precautions must be taken for patients requiring general anesthesia for surgery. Anesthesia procedures such as intubation, manual ventilation, and bronchoscopy, all generate a virus aerosol, and increase the risk of COVID-19 transmission [9]. In addition, airway surgery requires mitigation of viral aerosolization within the surgical field. Particularly, in cases with open-airway surgery, such as TBI repair, it is essential to protect healthcare workers from aerosol generation due to mechanical ventilation. ECMO can contribute to reducing aerosol exposure by avoiding intraoperative ventilation.

## Conclusions

The use of ECMO for traumatic TBI in a patient with COVID-19 facilitate surgical repair by eliminating the need for surgical field ventilation while contribute to prevent the spread of infection by reducing aerosol exposure.

## Supplementary Information

**Additional file 1: Figure S1.** Standard precautions for surgical treatment of COVID-19 patients in our hospital.

**Additional file 2: Table S1.** Perioperative management protocol for COVID-19 patients in our hospital.

## Data Availability

Not applicable.

## Abbreviations

TBI: Tracheobronchial injury
ECMO: Extracorporeal membrane oxygenation
PT: Prothrombin time
APTT: Activated partial thromboplastin time
FDP: Fibrin/fibrinogen degradation products
PCR: Polymerase chain reaction
ICU: Intensive care unit
POD: Postoperative day
ACT: Activated clotting time
