# Supplementary material for: Tracheal bifurcation repair for blunt thoracic trauma in a patient with COVID-19
Source: Surg Case Rep. 2023 Jun 15;9:108. doi: 10.1186/s40792-023-01695-8 (PMC10266956; doi:10.1186/s40792-023-01695-8)
Supplement: Supplementary file 2 — Additional file 2: Table S1. Perioperative management protocol for COVID-19 patients in our hospital. [file 40792_2023_1695_MOESM2_ESM.docx]

Table S1. COVID-19 patients’ surgical management

| All personnel in contact with the patient must wear personal protective equipment (N95 mask, disposable double gowns, double pair surgical gloves, protective goggles, disposable head caps, disposable shoe covers). |
| --- |
| COVID-19 patients should be transferred to a negative pressure operating room (OR) via the shortest route possible. |
| Once the patient has entered, the OR doors must always be closed. |
| Induce general anesthesia with intravenous anesthetics and rapid intubation (our patient was already intubated). |
| To minimize exposure to infected patients, surgeons should enter the OR after intubation. |
| Supply of materials to the OR during surgery should be minimal via the anteroom. |
| Following surgery, the OR and anteroom require sanitization and 40 minutes of ultraviolet radiation. |
